# Associations of food group and nutrient intake, diet quality, and meal sizes between adults and children in the same household: a cross-sectional analysis of U.S. households

**DOI:** 10.1186/1475-2891-10-131

**Published:** 2011-11-28

**Authors:** Jennifer L Zuercher, David A Wagstaff, Sibylle Kranz

**Affiliations:** 1 Department of Foods and Nutrition, Purdue University, 700 W. State Street, West Lafayette, IN 47906 USA; 2 Department of Health and Human Development, The Pennsylvania State University, N12A Henderson Building, University Park, PA 16802 USA

**Keywords:** child nutrition, nutrition survey, food intake, eating behavior, diet quality

## Abstract

**Background:**

One might assume that individuals living in the same household have similar dietary intakes of food groups and nutrients. However, the manner in which an adult's dietary intake affects children's food consumption, diet quality (defined as meeting intake recommendations), and meal sizes is understudied to date. The objective of this study was to estimate these relationships between minor children and the female or male head of household.

**Methods:**

Dietary intakes of one randomly selected child of each age group (2-5, 6-11, or 12-18 years old (n = 2,380)) and that of the female/male head of household ((HH), proxy for mother and father) using multiple 24-hour recalls from the Continuing Survey of Food Intake by Individuals (CSFII) 1994-1996 was coded to reflect food group and nutrient density (servings/grams per 1,000 kcal). Linear or logistic regression models were used to determine the association between intakes, whether individuals' diets trended toward meeting her/his intake recommendations, and whether individuals were in the highest quintile for food group densities at four distinct eating occasions (breakfast, brunch/lunch, supper/dinner, or other) in each subject group. Stata's survey commands were used to fit linear or logistic regression models and obtain adjusted regression coefficients or odds ratios.

**Results:**

Associations between food group/nutrient densities were significant but weak to moderate. Adults with diets that trended toward meeting their intake recommendations doubled the odds for children to have diets that trended toward meeting the recommendations; for many meals, adults consuming in the highest quintile for food group density predicted that children's intakes were also in the highest quintile.

**Conclusions:**

Female and male adults living in the same household significantly affect children's food group and nutrient intakes, diet quality, and meal sizes. There is an urgent need for in-depth analysis to elucidate the underlying mechanisms, especially for studies involving both the female and male HH.

## Background

Dietary intake is associated with a number of health outcomes of public health importance. A diet that meets recommendations for both food groups [1] and nutrients [2-4], that is associated with preventing disease and promoting optimal health, is considered a high-quality diet [5]. Further, increased diet quality has been associated with lower risk for childhood obesity [6,7] and other chronic diseases [8-10].

Although one might assume that the diets of household members are similar, this assumption is understudied. In families, for instance, in addition to possible genetic taste preferences, factors such as parent's level of education and socioeconomic status have been found to affect children's diets [11,12]. Family structure and familial issues can affect eating patterns that predict children's diet quality [13,14]. Research on the association between parental feeding practices and fruit and vegetable intakes in both American and Dutch children found that clusters of certain behavioral practices are associated with increased consumption [15,16]. Furthermore, children's intakes were found to be associated with the amount and type of foods served. That is, parents who reported that they ate all of their food at mealtimes were more likely to feed their children high-fat foods. More specifically, these parents were 30% more likely to serve fried foods at least three times per week during meals with their children and 80% less likely to remove the visible fat from foods during the cooking process [17]. Only a few large cross-sectional studies have investigated the direct relationship between parental and child eating, for instance between mother's and daughter's eating behavior [18,19]. Very little is known about the amount of food children consumed at meals or snacks and if the meal sizes were associated with parental eating. Overall, the generalization of results of studies on parent-child eating relationships is difficult due to small sample sizes, the studies being conducted in specific ethnic groups, and the investigations of a narrow range of nutrients [20].

To date, no published study has used a nationally representative sample of households to test the hypotheses that there is a strong positive association between the diets of female/male heads of household (HH) and a) children's food group and nutrient intakes, b) children's odds of consuming diets that trend toward meeting dietary intake recommendations, or c) the possible relationship between adult's and children's meal size. We addressed the latter question by estimating the association between the adult having a profoundly larger food group intake (top 20% of the population's intake) and the child having a profoundly larger food group intake. Although the findings from the present study were based on cross-sectional data and cannot demonstrate causal relations, we hope to provide sufficient evidence to spark an interest in further examination of the issues.

## Methods

The present CSFII analyses are based on publicly available secondary data that did not provide information that could be used to identify participants. Therefore, the IRB, which is charged with protecting people, does not consider these data "person data" and their study was exempt from review.

This study addressed three research questions: a) Are food group and nutrient intakes in adults and children living in the same household associated?, b) Are children more likely to consume diets that trend toward meeting their age-and-gender specific intake recommendations for food groups and nutrients if the female or male HH also consumes a diet that trends toward meeting her/his intake recommendations?, and c) Are children's odds to be a "big eater" higher when the female/male head of household has been identified as a "big eater" at the same eating occasion (i.e. meal type)?

These research questions were addressed using a sample of children 2-18 years old and the male and/or female head of household (HH). All individuals were participants in the Continuing Survey of Food Intake by Individuals (CSFII) 1994-1996 [21], a stratified, multistage, area probability sample of nationally representative data. Since it is not possible to determine if the mother or father of the child is in the data set, we used the female and male head of households as proxies, representing the female or male in charge. The HHs were identified in the survey by responses to the question "Who is the female (or male) in the household, who is in charge?" The response to this question was used to identify one female and/or male HH in each household. Unlike other approaches that limited the information of male HH to only those males who co-habitated with a female HH [22], we included all male HH (male only households: n = 335, female only n = 446, and both n = 940). Because more recent nationally representative data sets only provide information on one household member, they could not be used to investigate the present study's research questions regarding adult-child dyads. Thus, although the CSFII data are 15 years old, if one assumes that the basic relationships between adults and children in the same household have not changed during that time, the associations found in this study are still applicable today.

The present study included households with at least one child between 2 and 18 years old (n = 1,721 households with 3,150 children). Children were divided into three separate groups: preschoolers (2-5 year olds), school-age (6-11 year olds), and teenagers (12-18 year olds). When multiple children within the same age group resided in the same household, one child in that age group was randomly selected. Thus, 2-5-year-old children (n = 708), 6-11 year-old children (n = 836), and 12-18-year-old (n = 836) comprised the total sample (n = 2,380). The children not randomized into the study (n = 770) were excluded. This study design addressed the potential for cluster effects of individuals from the same household. We stratified all analyses by age group, including only the female or male HH and one child in each regression model for all six adult-child dyads.

Dietary intake data was obtained via one in-person 24-hour food recall and a second telephone recall collected three to ten days after the first recall but not on the same day of the week. The interview respondent reported diets of children who were younger than six years old. Children seven years and older provided their own diet information, assisted by an adult if necessary. Tippett and Cypel [23] provided a detailed explanation of the CSFII data collection procedures for dietary intakes.

For the first two research questions, dietary intake estimates of usual intake were based on the calculated two-day average food group and nutrient intakes in individuals with two reported days of intake (n = 2,254) and one day for those with only one day of intake (n = 126). The individuals with only one reported day of intake were not significantly different from those with two days of intake with respect to gender, ethnic, or income group.

Since the third research question was based on eating occasions, only data from the first 24-hour recall was used (n = 2,380). This approach was necessary because it is not possible to ascertain "usual intake" estimates for food group or nutrient intakes specific to an eating occasion. For instance, if an individual ate breakfast on the first day but skipped breakfast on the second day, only the first day would be included in the analysis (the arithmetic mean calculated from the intake of one day and the absence of intake on the second day would not be reflective of usual intake, i.e. (1 egg on day 1+ 0 eggs on day 2)/2 = 1/2 egg each day - which does not represent the person's eating pattern).

Eating occasions were recorded based on the respondent's description. Similar to the manner with which other researchers have used this variable [24], intakes were categorized by the survey respondent as breakfast, brunch, lunch, dinner, supper, food/beverage, infant feeding, extended eating, and other. Because the present study only included children who were between the ages of 2 and 18, the category "infant feeding" (n = 57) was not included. Since both, "brunch" and "lunch" were reported between 11 am and 2 pm, we combined those two categories. Similarly, we combined "supper" and "dinner". We also combined the infrequent categories "food or beverage" and "other" eating occasions. Following the consolidation, the final categories for "eating occasion" and their corresponding frequencies for children and adults were breakfast (n = 36,763)), brunch/lunch (n = 36,809), supper/dinner (n = 50,586), and "other, food/beverage" (n = 1,980).

Total energy in kcal, the number of servings from six Food Guide Pyramid [25] food groups (fruits, vegetables, total grain, whole grain, milk/dairy, meat), and consumption of macronutrients and micronutrients in grams and micrograms, as appropriate, for total fat, saturated fat, unsaturated fatty acids (linoleic and linolenic acid), protein, carbohydrates, cholesterol, dietary fiber, added sugar, iron, calcium, vitamins A, B12, and C, and folate were calculated. Dietary recalls that were coded in the data set as "reliable" but showed average energy consumptions with implausible values (< 500 kilocalories (kcal) of total energy intake reported per day) were excluded [26]. To update the Food Guide Pyramid serving sizes to the corresponding MyPlate portion sizes, consumption was converted to represent the serving sizes cups and ounces of the MyPlate food groups. Means and standard errors for food group and nutrient intakes were calculated and expressed in energy-adjusted terms (servings of food groups or grams/micrograms of nutrients per 1,000 kcal of total energy consumed). Binary indicator variables were created to identify the individuals whose dietary intake was below the recommended consumption level (= 0) or those who trended toward meeting the recommended level (= 1) for total energy intake (Estimate Energy Requirements (EER)), macronutrients (Acceptable Macronutrient Distribution Range (AMDR)) or micronutrients (Estimated Average Requirement (EAR)) and Adequate Intake (AI) for dietary fiber and calcium of the Dietary Reference Intakes (DRI). Total food group density (fruit, vegetables, total grain, whole grain, milk/dairy, meat) per eating occasion (breakfast, brunch/lunch, dinner/supper, other food/beverage) was calculated to represent the energy adjusted amount of intake from each food group consumed at each eating occasion. Food group densities were ranked for each of the five population groups (female HH, male HH, children ages 2-5, 6-11, 12-18 year olds). To examine if "big eaters" clustered within families, we estimated the odds that the child's intake fell in the highest quintile of food group consumption per eating occasion if the female or male HH's intake fell in the highest quintile. This measure of analysis was chosen because grams of food consumed or calories consumed are variables that do not account for the person's intake of other foods and body size, whereas number of servings per 1,000 kcal consumed (food group density) accounts for both of these cofactors of intake. Thus, we defined "big eaters" as those individuals who consumed within the highest 20% of total food group density consumption (highest quintile) within each population group (preschoolers, school-age children, adolescents, female HH, or male HH) and eating occasion (breakfast, brunch/lunch, supper/dinner, other food/beverages).

Descriptive statistics (proportions, means and standard errors) were obtained. Linear and logistic regression models for complex sample survey data were fit to explore a) the association between the food group and nutrient densities reported for the female and/or male HH and for the children in the same household, b) the odds that the child consumed a diet that trended toward meeting his/her age- and gender-specific dietary intake recommendations for food groups and nutrients when the female/male HH consumed a diet that trended toward meeting her/his corresponding intake recommendation and, c) the odds that the child's intake fell in the highest quintile for food group density of the eating occasions if the female or male HH's intake fell in the highest quintile of the same food group and eating occasion.

To control for socio-demographic variables, age, gender, race, country of origin, years of education, employment status, daycare/school attendance, and total household size and income were examined. Education (years of schooling) was categorized as less than high school (< 12 years of school), high school (12 years of school), and more than high school (> 12 years of school). Employment status was categorized as employed or not employed. Total household income was used as a continuous variable. Its skew of the distribution was addressed by log transformation. To describe the sample, income was also categorized as: less than or equal to 1.3 times the poverty income ratio (PIR) (food stamp eligible), 1.31 to 3.5 times the PIR (medium income), and more than 3.5 times the PIR (high income). To capture cultural differences, a study participant's race (black, white and other) and Hispanic origin (from the Mexican, Puerto Rican, Cuban, other Spanish subgroups, or not) variables were re-coded into Non-Hispanic white, Non-Hispanic black, Non-Hispanic Asian, Non-Hispanic other, Mexican-American, and other Hispanic. Binary dummy variables were subsequently generated for ethnicity (not Hispanic, Hispanic) and HH's educational level (less than high school, high school graduate, more than high school graduate).

Twenty-one separate linear regressions in the six adult-child dyads were modeled for the six food groups and 15 nutrients under study. Linear regression models were fit to calculate the associations between food group and nutrient densities in the children's and adult head of household's diets; logistic regression models for complex sample survey data were used to obtain adjusted odds ratios for the odds of children consuming diets that trended toward meeting intake recommendations for food groups and nutrients if the female/male HH consumed a diet that trended toward her/him meeting her/his; and logistic regression models were used to obtain adjusted odds ratios for the odds of children to be in the highest food group density intake quintile for an eating occasion if the female/male HH was in the highest quintile. Regression models were not fitted if one of the four cells in the 2 × 2 table calculating the odds ratios had a sample size of < 10 subjects. Models controlled for household size, age, education level, ethnicity, and income. All statistical methods were executed in Stata Statistical Software: Release 9.0 (Stata Corporation, College Station, TX, USA) using complex sample survey routines that account for the CSFII's unequal selection probabilities, stratification, and clustering, and maintain the nationally representative character of the data.

## Results

Table 1 describes selected characteristics of the households while Table 2 indicates the characteristics of the children in the sample. Linear regression coefficients that describe relations between food group and nutrient densities of the diets of the female HH and the children (Table 3) indicate significant, weak to moderate positive associations between the intakes. The strongest association was observed for meat density in preschoolers' diets (β = 0.34) and the weakest association was for iron in teenagers (β = 0.14). Generally, associations were slightly stronger in the preschool and school-age children than in the teenagers. The associations between intakes of the male HH and his children were also positive but weak to moderate (Table 4). Here, the strongest association was observed for fruit density between the diets of preschoolers and the male HH (β = 0.40). The only non-significant associations were observed for Vitamin B12 densities in the diets.

**Table 1:** Characteristics of the sample households and heads of households (in percent)

		Head of Household			
Characteristic	Value	Female N = 446	Male N = 335	Female and Male N = 940	
				Female	Male
Age (Years)	14-18	0.0	1.5	0.2	0.4
	19-30	20.2	16.5	17.7	13.9
	31-50	64.8	71.2	74.3	72.0
	> 50	15.0	10.8	7.8	13.7
					
Race/Ethnicity	Non-Hispanic White	52.5	68.5	73.5	
	Non-Hispanic Black	29.2	9.9	9.1	
	Non-Hispanic Asian	1.4	2.7	3.3	
	Non-Hispanic Other	2.7	1.5	0.9	
	Mexican-American	5.2	7.2	7.6	
	Other Hispanic	9.2	10.2	5.6	
					
Education	Less than High School	22.2	18.2	12.4	13.9
	High School	37.1	32.5	38.2	35.0
	More than High School	40.7	49.2	49.4	51.2
					
Employed		62.8	86.5	66.4	90.2
					
Poverty Income	< 1.3	50.7	100.0	18.6	
Ratio	1.31-3.5	31.6		46.7	
	> 3.5	17.7		34.7	
					
% female children		64.6	60.4	68.4	
					
# Children in household	1	48.0	55.6	40.1	
	2	32.1	32.4	37.7	
	3	13.9	7.8	15.2	
	4	3.8	3.3	5.3	
	5-8	2.2	0.9	1.7	
					
Age of children	2-5	26.1	38.3	25.6	
	6-11	34.8	35.5	35.2	
	12-18	39.1	26.3	39.2	

**Table 2:** Characteristics of the Children in the Household

		Age group		
		2-5 N = 708	6-11 N = 836	12-18 N = 836
Characteristic	Value			
Average age (yearsn ± SD)		3.5 ± 1.1	8.6 ± 1.7	14.8 ± 1.9
Sex (%)	Girls	49.6	48.8	50.5
Race/Ethnicity (%)	Non-Hispanic White	60.5	62.1	65.9
	Non-Hispanic Black	15.8	15.7	14.5
	Non-Hispanic Asian	2.3	2.9	3.0
	Non-Hispanic Other	3.1	2.8	1.2
	Mexican-American	9.7	9.1	6.8
	Other Hispanic	8.6	7.5	8.6

**Table 3:** Linear regression coefficients for selected food group and nutrient densities between children the female head of household (HH)*

	**2-5 year olds**	***P***	**6-11 year olds**	***P***	**12-18 year olds**	***P***
	
Meat density	0.34 ± 0.03	< 0.001	0.30 ± 0.03	< 0.001	0.28 ± 0.03	< 0.001
Grains density	0.28 ± 0.06	< 0.001	0.29 ± 0.04	< 0.001	0.28 ± 0.04	< 0.001
Fruit density	0.22 ± 0.09	0.020	0.23 ± 0.07	0.001	0.21 ± 0.05	< 0.001
Vegetable density	0.20 ± 0.03	< 0.001	0.19 ± 0.03	< 0.001	0.19 ± 0.02	< 0.001
Milk density	0.26 ± 0.07	< 0.001	0.32 ± 0.04	< 0.001	0.30 ± 0.03	< 0.001
Cholesterol density	0.27 ± 0.04	< 0.001	0.27 ± 0.04	< 0.001	0.25 ± 0.03	< 0.001
Fiber density	0.21 ± 0.04	< 0.001	0.23 ± 0.03	< 0.001	0.19 ± 0.03	< 0.001
Iron density	0.20 ± 0.04	< 0.001	0.15 ± 0.03	< 0.001	0.14 ± 0.02	< 0.001
Calcium density	0.30 ± 0.06	< 0.001	0.27 ± 0.05	< 0.001	0.25 ± 0.04	< 0.001
Folate density	0.16 ± 0.03	< 0.001	0.16 ± 0.03	< 0.001	0.16 ± 0.03	< 0.001
Vit. C density	0.18 ± 0.05	0.001	0.18 ± 0.04	< 0.001	0.17 ± 0.03	< 0.001
Vit. A density	0.23 ± 0.02	< 0.001	0.24 ± 0.03	< 0.001	0.20 ± 0.03	< 0.001
Vit. B12 density	0.22 ± 0.02	< 0.001	0.22 ± 0.04	< 0.001	0.16 ± 0.05	0.001

*Models controlled for household size, age, education level, ethnicity, and income.

** Food group and nutrient density: servings of food group or gram/milligram of nutrient per 1,000 kcal consumed

**Table 4:** Linear regression coefficients for selected food group and nutrient densities between children the male head of household (HH)*

	**2-5 year olds**	***P***	**6-11 year olds**	***P***	**12-18 year olds**	***P***
	
Meat density	0.20 ± 0.03	< 0.001	0.22 ± 0.03	< 0.001	0.25 ± 0.03	< 0.001
Grains density	0.30 ± 0.05	< 0.001	0.29 ± 0.04	< 0.001	0.32 ± 0.03	< 0.001
Fruit density	0.40 ± 0.09	< 0.001	0.35 ± 0.06	< 0.001	0.31 ± 0.04	< 0.001
Vegetable density	0.19 ± 0.03	< 0.001	0.19 ± 0.04	< 0.001	0.19 ± 0.03	< 0.001
Milk density	0.21 ± 0.07	0.008	0.22 ± 0.05	< 0.001	0.24 ± 0.04	< 0.001
Cholesterol density	0.25 ± 0.05	< 0.001	0.24 ± 0.03	< 0.001	0.21 ± 0.03	< 0.001
Fiber density	0.22 ± 0.04	< 0.001	0.23 ± 0.04	< 0.001	0.23 ± 0.03	< 0.001
Iron density	0.20 ± 0.04	< 0.001	0.23 ± 0.06	< 0.001	0.20 ± 0.04	< 0.001
Calcium density	0.23 ± 0.06	< 0.001	0.25 ± 0.05	< 0.001	0.27 ± 0.05	< 0.001
Folate density	0.19 ± 0.04	< 0.001	0.20 ± 0.04	< 0.001	0.19 ± 0.03	< 0.001
Vit. C density	0.19 ± 0.07	0.008	0.28 ± 0.04	< 0.001	0.28 ± 0.03	< 0.001
Vit. A density	0.35 ± 0.10	0.002	0.22 ± 0.07	0.004	0.23 ± 0.06	0.001
Vit. B12 density	0.29 ± 0.18	0.124	0.33 ± 0.19	0.094	0.32 ± 0.19	0.099

*Models controlled for household size, age, education level, ethnicity, and income.

** Food group and nutrient density: servings of food group or gram/milligram of nutrient per 1,000 kcal consumed

**Figure 1:**
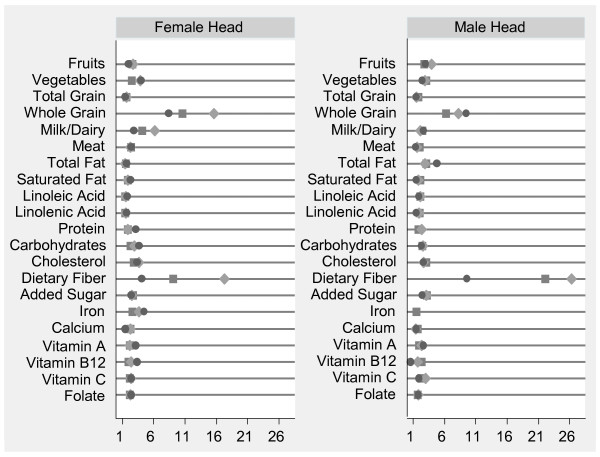
**Adjusted odds ratio for children to consume a diet that trends toward meeting intake recommendations when female/male HH consumed a diet that trended toward meeting her/his recommendation**. Circle represents 2-5 year olds, Large diamond represents 6-11 year olds, Square represents 12-18 year olds, Models correct for household size, head of household's (HH) age, HH's education level (less than high school (referent), high school, more than high school), ethnicity (not Hispanic or Hispanic), and household income. Results for milk, whole grains, and dietary fiber must be interpreted with cautions due to excessively large 95% CIs. ORs for consuming a diet that trends toward meeting protein intake in preschoolers or iron recommendations in preschoolers and school-age children when the male HH consumed a diet that trended toward meeting his protein or iron intake recommendation could not be computed due to perfect prediction. Results are significant at P < 0.05 except for the odds of the female HH consuming a diet that trended toward meeting her recommendations and preschoolers consuming a diet that trends toward meeting theirs for total grains, protein, dietary fiber, calcium, iron, vitamin C, and vitamin B12 as well as school-age children for protein, iron and vitamin B12 or the male HH consuming a diet that trends toward meeting his recommendations and preschoolers consuming a diet that trends toward meeting theirs for total grains, saturated fat, dietary fiber, vitamin c and vitamin B12 as well as school-age children consuming a diet that trends toward meeting the recommendation for vitamin B12 recommendation and in teenagers the odds for consuming a diet that trends toward meeting the iron recommendation.

Figure 1 provides a succinct summary of the odds ratios calculated with the covariate-adjusted logistic models for complex sample survey data (odds ratios, p-values, and 95% confidence intervals for all food groups and nutrients in the six adult/child dyads are not reported but are available upon request). Some calculated odds ratio estimates were problematic due to sparse data; in several instances, one of the two cell frequencies used to obtain the odds ratio's denominator was very small, resulting in an excessive standard error estimate. For example, the dietary fiber intake recommendation only trended toward being met by n = 9 of the 12-18 year old children and n = 30 of the female HHs among the 600 child/female HH dyads included in this analysis. Thus, in the model calculating children's odds of consuming a diet trending toward meeting the dietary fiber recommendation when the female HH consumed a diet that trended toward meeting her intake recommendation, the "yes-yes" cell had very few subjects. The same "small cell count" problem was observed for whole grain and dairy intake in preschoolers when the female HH consumed a diet that trended toward her meeting recommendations for these food groups.

Generally, most odds ratios were larger than one, indicating that children's diets were positively related to the HHs consuming diets that trended toward meeting her/his intake recommendation. More specifically, the odds of children to consume diets that trend toward meeting the intake recommendations when the female HH consumed a diet that trended toward meeting her recommendation were significant for all but one food group (total grains) and several nutrients in preschoolers (protein, dietary fiber, calcium, iron, vitamin C, and vitamin B12) and school-age children (protein, iron, and vitamin B12). Similarly, children's odds of consuming a diet that trends toward meeting food group and nutrient intake levels when the male HH consumed a diet that trended toward meeting his intake recommendations were significant except in preschoolers (total grains, saturated fat, dietary fiber, vitamin C, and vitamin B12), in the school-age children (vitamin B12), and in teenagers (protein and iron). We were not able to compute odds ratios for consuming a diet that trends toward meeting the protein intake recommendation in preschoolers or the iron recommendation in preschoolers and school-age children when the male HH consumed a diet that trended toward meeting his recommendation due to the occurrence of a zero cell frequency in one of the off-diagonal cells (the "prefect prediction" problem).

The odds of children to consume meals or snacks that were ranked in the highest quintile of food group density when the adult's (female or male head in a female- and male headed household) consumption was in the top 20% of food group density are reflected in Table 5 (female HH) and Table 6 (male HH). Due to the limited number of households with one female or male head of household (i.e. single-parent households) and the stratification by four distinct eating occasions, many cells of the 2 × 2 table used to estimate crude odds ratios were too small to provide reliable estimates. In those conditions where sample sizes did not restrict the ability to statistically determine associations, children's odds of being a "big eater" of certain food groups were positive and significant at many eating occasions and meals. For instance, the odds of children being in the highest quintile of grain density were increased in all age groups for at least one meal occasion. Specifically, children were twice as likely to have high grain consumption at brunch/lunch and dinner/supper when the female HH consumed a meal with high grain density at these meals. Male HH with high grain intake at breakfast predicted children to also have high grain intake at breakfast. The odds of having high vegetable density at dinner/supper was more than five-fold in preschoolers (OR = 5.7) and adolescents (OR = 5.9) when female HH had high vegetable consumption at that meal; likewise the odds for high meat density at dinner/supper was much higher in preschoolers (OR = 6.5) and in school-age children (OR = 13.3). Overall, no negative associations (odds ratios of less than zero) were observed.

**Table 5:** Odds of children to have food group density in the highest quintile by eating occasion, if intakes of female head of household (HH) were in the highest quintile

Meal	Food	Age Group					
Occasion	Group Density	2 - 5		6 - 11		12 - 18	
	Grains	1.28	(0.84, 1.72)	1.54	(0.98, 2.10)	1.75	(1.13, 2.37) *
	Fruit	--------		0.82	(0.23, 1.41)	1.63	(0.79, 2.47)
Breakfast	Vegetables	--------		--------		--------	
	Milk/Dairy	1.20	(0.44, 1.96)	1.89	(1.03, 2.75) *	3.23	(1.85, 4.61) *
	Meat	3.17	(0.69, 5.65)	2.68	(0.81, 4.55)	2.55	(0.80, 4.30)
	
	Grains	1.76	(1.12, 2.40)*	3.23	(2.05, 4.41) *	1.87	(1.23, 2.51) *
	Fruit	--------		3.20	(1.49, 4.91) *	2.83	(0.73, 4.93)
Brunch/Lunch	Vegetables	2.69	(1.41, 3.97) *	2.73	(1.42, 4.04) *	1.20	(0.79, 1.61)
	Milk/Dairy	1.68	(0.83, 2.53)	3.14	(1.90, 4.38) *	1.70	(0.77, 2.63)
	Meat	2.04	(0.84, 3.24)	1.71	(0.98, 2.44)	3.01	(1.79, 4.23) *
	
	Grains	1.91	(1.18, 2.64) *	2.93	(1.92, 3.94) *	1.79	(1.08, 2.50) *
	Fruit	--------		3.80	(1.38, 6.22) *	--------	
Dinner/Supper	Vegetables	5.70	(3.41, 7.99) *	3.95	(2.43, 5.47) *	5.93	(3.37, 8.49) *
	Milk/Dairy	2.43	(0.97, 3.89)	2.65	(1.59, 3.71) *	6.68	(3.31, 10.05) *
	Meat	6.45	(2.68, 10.22) *	13.34	(7.03, 19.65) *	1.43	(0.83, 2.03)
	
	Grains	--------		4.85	(1.32, 8.38) *	3.79	(1.54, 6.04) *
	Fruit	--------		3.19	(1.22, 5.16) *	--------	
Food/Beverage	Vegetables	--------		--------		--------	
	Milk/Dairy	--------		--------		--------	
	Meat	--------		--------		--------	

* OR significant

**Table 6:** Odds of children to have food group density in the highest quintile by eating occasion, if intakes of male head of household (HH) were in the highest quintile

Meal	Food	Age Group					
Occasion	Group Density	2 - 5		6 - 11		12 - 18	
	Grains	2.87	(1.78, 3.96) *	1.71	(1.04, 2.38) *	2.60	(1.76, 3.44) *
	Fruit	3.38	(1.45, 5.31) *	5.16	(3.87, 6.45) *	1.92	(0.62, 3.22)
Breakfast	Vegetables	--------		--------		--------	
	Milk/Dairy	2.23	(1.14, 3.32) *	--------		2.43	(1.31, 3.55) *
	Meat	--------		4.84	(0.97, 8.71)	1.41	(0.49, 2.33)
	
	Grains	1.76	(1.18, 2.34) *	1.01	(0.57, 1.45)	1.88	(1.37, 2.39) *
	Fruit	--------		--------		1.87	(0.44, 3.30)
Brunch/Lunch	Vegetables	1.40	(0.85, 1.95)	1.99	(1.29, 2.69) *	0.65	(0.36, 0.94)
	Milk/Dairy	0.93	(0.41, 1.45)	3.35	(1.42, 5.28) *	1.32	(0.47, 2.17)
	Meat	1.09	(0.46, 1.72)	1.77	(1.00, 2.54)	2.01	(1.19, 2.83) *
	
	Grains	1.36	(0.85, 1.87)	3.59	(2.48, 4.70) *	4.54	(2.80, 6.28) *
	Fruit	--------		--------		7.09	(2.07, 12.11) *
Dinner/Supper	Vegetables	3.81	(2.10, 5.52) *	3.21	(1.89, 4.53) *	4.40	(2.63, 6.17) *
	Milk/Dairy	2.25	(1.19, 3.31) *	4.43	(2.93, 5.93) *	3.48	(1.43, 5.53) *
	Meat	2.32	(1.28, 3.36) *	4.11	(2.09, 6.13) *	1.85	(1.26, 2.44) *
	
	Grains	--------		0.69	(0.37, 1.01)	--------	
	Fruit	1.96	(0.43, 3.49)	--------		--------	
Food/Beverage	Vegetables	--------		--------		--------	
	Milk/Dairy	--------		--------		--------	
	Meat	--------		--------		--------	

* OR significant

## Discussion

Children learn many important behaviors and skills from the adults with whom they live, such as attitudes towards food and feeding, and their feeding style (i.e. parent centered vs. child centered, using food as a reward, etc.), which influence food preference [27]. In-depth literature searches have yielded only weak results for a causal relationship between parental eating practices or feeding styles and children's eating, in part, because most studies were cross-sectional or limited by their sample characteristics [28].

To establish a causal relationship between parental and child eating habits, longitudinal studies are indicated. Until the data from such studies become available, researchers will have to use the data from repeated, cross-sectional studies to advance the understanding of the role that dietary intakes play in the diets of adults and their children. With the present study, we used data from a nationally representative sample of noninstitutionalized adults and children living in U.S. households to investigate associations between the dietary intake of selected food groups and nutrients of adults and children. Because the data set did not permit us to identify parent-child dyads, we used the female and male head of household as proxies for the child's mother and father. The underlying assumption of this method is based on the premise that adult role models within the household affect children's eating behavior, even if the adult is not the biological parent of the child.

Our analyses addressed three questions: "Are food group and nutrient intakes in adults and children living in the same household associated?", "Are children more likely to consume diets that trend toward meeting their age-and-gender specific intake recommendations for selected food groups and nutrients if the female/male HH consumes a diet that trends toward meeting her/his intake recommendations?", and "Are children's odds to be a "big eater" higher when the female/male head of household has been identified as a "big eater"?".

To answer the first question, we fit linear regression models to estimate regression coefficients that reflected the direction and strength of the association between the density of six food groups and 15 nutrients in the diets of children and the diets of the female/male HH. Results of this study are corroborated by other studies examining the association between parental and child diets. These studies were predominantly from small samples and/or non-generalizable populations [29]. However, a large study that corroborates, in part, our results was indeed conducted in the same data set [30]. Unlike Wang et al., we focused our examination on the food group and nutrient densities in the diet, rather than the absolute amount of food consumed. This approach led to stronger association estimates for intakes under study, indicating the importance of considering total energy consumption when examining food group and nutrient intakes. Overall, although research indicates that the association between food group and nutrient intakes of adults and children are moderate at best, or findings are limited to specific race/ethnicity groups, results indicate the importance of including parental nutrition interventions to improve children's diets and health. For instance, the finding that mother's beverage choice had a formative environmental influence on daughter's beverage choice [19]; if mothers drank more milk and less soft drinks, the daughters tended to drink more milk and less soft drinks. Consequently, the authors suggested that encouraging mothers to consume more milk might lead to children's increased milk intake. Some studies report data indicating that parent's fruit and vegetable consumptions were strongly and positively related to their children's fruit and vegetable intake [31,32].

Question two and three were addressed by fitting logistic regressions, estimating the odds of the child consuming a diet that trends toward meeting his/her recommended intake level when the female/male HH consumed a diet that trended toward her/him meeting her/his recommendation (Question 2) and estimating the odds of the child being ranked in the highest quintile of food group density per eating occasion when the female/male HH was in the highest quintile for the same food group density at the same eating occasion (Question 3).

Although the associations between the adult's and child's food group or nutrient consumption was not very strong, the child's odds of consuming a diet that trends toward allowing her/him to meet her/his age- and gender-specific food group recommendations were doubled for most food groups and nutrients under study when the female or male HH consumed a diet that trended toward allowing her/him to meet her/his intake recommendation. To our knowledge, this finding has not been reported previously. Interestingly, children's odds for consuming a diet that trends toward meeting their fruit and vegetable intake recommendations were at least doubled when the HH consumed a diet that trended toward meeting her/his recommended levels. This observation is consistent with the report that father's fruit consumption appeared to have the strongest impact on children's intake [33], which might be based on the fact that vegetables are more likely consumed during meals while fruits might be eaten frequently as a snack. Father's choice of fruit for a snack may contribute to children making the same choice. It is noteworthy to point out that parental feeding style is differential between fruit and vegetable feeding in that feeding vegetables has been found to occur in a negative context, while feeding fruits occurs in a positive context [16]. If or how this factor affects the female or male adult in the household differentially remains to be studied.

More research on the amounts of foods consumed by children at eating occasions throughout the day is needed. Studies which have used nationally representative data have shown that children are eating more frequently and that portion sizes have increased over the past 30 years [34]. One study indicated that children's body weight status and familiarity with the person in whose company they eat affects the amount of food consumed [35].

Studies have shown that increased portion sizes are associated with larger amounts of food consumed [36,37]. In contrast to animals, people may chose to eat more food in spite of the physiological responses to food ingestion related to the psychological construct of expectation or the need to overwrite the post-prandial signaling [38]. This concept does not seem to apply to very young children [39]. The potential effect [40] of removing visual cues of the meal and the amount of food consumed has been explored to some extent [41]. However, randomized controlled studies in children are needed to affirm or refute these findings in the pediatric population. A rather provocative approach to research the effect of visual cues, or absence thereof, on food intake was to remove all visual cues by having the study participants eat in complete darkness. Results showed that young adults consumed more when served a larger meal independent of their ability to see their food [40]. Thus, although it can be assumed that the amount of food served affects meal size, the effect of eating in the presence of a parent who consumes large amounts of food on children's meal size requires further study. Our data indicates the potential for such a relationship. While one is not able to ascertain information on family meals or whether children ate in the presence of an adult from this data set, it is feasible to assume that children observe their parents' eating behavior even if meals are not always taken together. Although the days of dietary intake collection were likely not the same and we fail to see whether eating large portions occurs simultaneously, we were able to show that children are more likely to be big eaters of some food groups at certain eating occasions, such as having a large breakfast, when the female or male HH was a big eater of the same food group at that meal. This finding has not been reported previously and additional analysis as well as longitudinal studies to determine the predictors of having large meal sizes are indicated.

Our data indicate that the role of the male HH on children's dietary intakes has been underestimated. Unfortunately, this aspect of child nutrition has been much neglected. The traditional approach of focusing on the female HH as the person who determines what family members eat when they share meals has affected study designs [42]. Male HH were either not included or only included when they lived in households with a female HH, which might have led to the conclusion that the female HHs had a stronger effect on children's diets than her male counterparts [22]. Since studies that have enrolled the male HH as an active participant are either small with respect to sample size or scope, the results of our study need to be confirmed by further studies.

Our study was limited by a number of factors. First, we used the generally accepted venue of assigning the female and male HH as proxies for a child's mother and father. Thus, the interpretation of our data is limited to the associations of children's diets with that of the female or male head of household - we did not evaluate the effect of a mother's or father's intakes. We also could not determine or account for whether or not meals were consumed together (i.e. if the child and the adult ate in close proximity to one another). Furthermore, the diets of children were reported for foods consumed at home as well as away from home. This is a recurrent issue in using 24-hour recalls to gather information on diets of children younger than six years old. Many of the intakes reported are best possible estimates of the reporting adult and not based on actual observation of the child's intake. The use of 24-hour recalls to estimate usual intake in and of itself is a limitation. The use of two recalls completed over a short (roughly one week) period by each individual serves as a substitute for "usual intake" while in reality, the use of multiple recalls over time would provide a more accurate view of usual intake. Still, this is the accepted method for estimating usual intake in populations.

Children become more independent as they get older and they consume more foods away from home. Reports on whether children maintain the intake patterns adopted at a young age or when they are eating in the presence of the adult role model compared to when they are eating away from home and/or in the absence of the adult are not available to date. One can assume that with increasing age, an individual's diet is influenced by factors other than parental eating behavior factors other than the presence of adults affect children's diets. School and social environments, including peer influence, vending machine accessibility, and fruit and vegetable availability have a great impact on children's intakes [43]. In our study, approximately three quarters of the children were six years or older and thus have many opportunities for eating outside of the home. In-depth research is needed to examine the optimal entry points of nutrition interventions to improve diet quality in children who have become nutritionally independent.

In contrast to studies exploring children's dietary intake habits during role play [44], our findings indicated only moderate associations between adults' and children's diets but a strong association between adults' and children's diet quality when the latter was expressed as trending toward meeting dietary intake recommendations. This observation might be evidence for an underlying mechanism that reflects a threshold. Certainly, in-depth longitudinal research on this question is needed. Especially valuable would be the study of behavioral mechanisms underlying the development of food intake patterns of children and how they relate to children's age, gender, relationship with adults in the house, and their level of independence. Furthermore, future research may want to examine whether parental eating behavior may change as an adaptation to a child's being overweight or obese (c.f. Benton et al.[27]).

## Conclusion

Our study resulted in a number of practical new findings. First, female and male head of household's intakes were significant predictors of children's odds to consume a diet that trends toward meeting dietary intake recommendations for food groups and nutrients. Thus, fathers might have an effect on children's diets that is at least as strong as that of the mother. Also, parents consuming diets that led them to be ranked in the highest 20% of intake at an eating occasion resulted in many of the children being ranked in the same category. Thus, parental modeling of food intake might include the food groups chosen at the meal as well as the meal size.

## Abbreviations

HH: Head of household; CSFII: Continuing Survey of Food Intake by Individuals; EER: Estimated Energy Requirements; AMDR: Acceptable Macronutrient Distribution Range; EAR: Estimated Average Requirement; DRI: Dietary Reference Intakes; PIR: Poverty Income Ratio; OR: Odds Ratio

## Competing interests

The authors declare that they have no competing interests.

## Authors' contributions

SK conceived the study, led the project, and contributed to the writing of the manuscript. JLZ reviewed the literature and contributed to the writing. DAW conducted the analysis under SK's direction and contributed to the writing. All authors have approved this manuscript.

## Authors' information

JLZ holds a PhD in Nutrition with a focus on Interventions and Policy from UNC Chapel Hill. This work was completed as part of a Post Doctoral Fellowship at Purdue University. SK is a trained Nutrition Epidemiologist from UNC Chapel Hill with a number of publications using nationally representative data. Her main research focus is on children's intake behavior and diet quality. SK is an Associate Professor and Director of the Coordinated Program in Dietetics in the Department of Nutrition Science at Purdue University. DAW is a Research Technologist with the HHD Consulting Group, College of Health and Human Development, The Pennsylvania State University. One of his research interests is the analysis of complex sample survey data.
